# Predicting and preventing ovarian hyperstimulation syndrome (OHSS): the need for individualized not standardized treatment

**DOI:** 10.1186/1477-7827-10-32

**Published:** 2012-04-24

**Authors:** Klaus Fiedler, Diego Ezcurra

**Affiliations:** 1 Kinderwunsch Centrum München (KCM) (Fertility Center Munich), Lortzingstr. 26, D-81241, Munich, Germany; 2 Merck Serono S.A. – Geneva (an affiliate of Merck KGaA, Darmstadt, Germany), 9 Chemin des Mines, Geneva, CH-1202, Switzerland

**Keywords:** Ovarian hyperstimulation syndrome, Individualized controlled ovarian stimulation, Risk factors, Vascular endothelial growth factor, Prevention

## Abstract

Ovarian hyperstimulation syndrome (OHSS) is the most serious complication of controlled ovarian stimulation (COS) as part of assisted reproductive technologies (ART). While the safety and efficacy of ART is well established, physicians should always be aware of the risk of OHSS in patients undergoing COS, as it can be fatal. This article will briefly present the pathophysiology of OHSS, including the key role of vascular endothelial growth factor (VEGF), to provide the foundation for an overview of current techniques for the prevention of OHSS. Risk factors and predictive factors for OHSS will be presented, as recognizing these risk factors and individualizing the COS protocol appropriately is the key to the primary prevention of OHSS, as the benefits and risks of each COS strategy vary among individuals. Individualized COS (iCOS) could effectively eradicate OHSS, and the identification of hormonal, functional and genetic markers of ovarian response will facilitate iCOS. However, if iCOS is not properly applied, various preventive measures can be instituted once COS has begun, including cancelling the cycle, coasting, individualizing the human chorionic gonadotropin trigger dose or using a gonadotropin-releasing hormone (GnRH) agonist (for those using a GnRH antagonist protocol), the use of intravenous fluids at the time of oocyte retrieval, and cryopreserving/vitrifying all embryos for subsequent transfer in an unstimulated cycle. Some of these techniques have been widely adopted, despite the scarcity of data from randomized clinical trials to support their use.

## Background

There has been a rapid increase in the number of couples receiving treatment for infertility with assisted reproductive technology (ART) in recent years [1]. While there is robust evidence supporting the efficacy and safety of ART, it is important to be aware of the risks, the most serious of which is ovarian hyperstimulation syndrome (OHSS). OHSS is a rare, iatrogenic complication of controlled ovarian stimulation (COS). Severe OHSS occurs in approximately 1.4 % of all cycles [2], affecting approximately 6020 patients per year in the United States and Europe [3]. The mortality risk is estimated to be 1 in 450000 to 500000 cases [4].

### Pathophysiology and symptoms of OHSS

**Figure 1:**
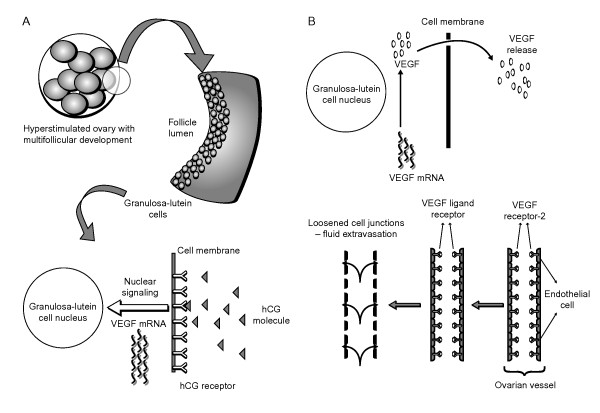
**The pathogenesis of OHSS.** Human chorionic gonadotropin (hCG) stimulates a high number of granulosa-lutein cells leading to the increased production of vascular endothelial growth factor (VEGF) mRNA (Figure 1A); VEGF receptor-2 (VEGFR-2) mRNA production in the granulosa-lutein and endothelial cells is also increased in response to hCG. High amounts of VEGF are produced and released from the granulosa-lutein cells and bind to VEGFR-2 on the endothelial cell membranes. Downstream signaling augments vascular permeability (Figure 1B). Adapted from Soares et al [7].

OHSS is an exaggerated response to COS characterized by the shift of protein-rich fluid from the intravascular space to the third space, mainly the abdominal cavity that occurs when the ovaries become enlarged due to follicular stimulation [5]. This shift in fluid is due to increased vascular permeability in response to stimulation with human chorionic gonadotropin (hCG) [5]. Prostaglandins, inhibin, the renin-angiotensin-aldosterone system and inflammatory mediators have all been implicated in the aetiology of OHSS [6]; however, vascular endothelial growth factor (VEGF) has been identified as the major mediator (Figure 1) [5]. The expression of VEGF and VEGF receptor 2 (VEGFR-2) mRNA increases significantly in response to hCG, and peak levels coincide with maximum vascular permeability [5].

The clinical manifestations of OHSS reflect the extent of the shift of fluid into the third space and the resulting hemoconcentration due to intravascular volume depletion. Symptoms range from mild abdominal distention due to enlarged ovaries alone or with an accompanying fluid shift into the abdomen, to renal failure and death as a result of hemoconcentration and reduced perfusion of organs such as the kidneys, heart and brain (Table 1) [5,8]. Indeed, as the severity of OHSS increases, so does the number of organs affected [8].

**Table 1:** **Classification of OHSS symptoms**[5] (adapted from Navot et al [9])

**OHSS stage**	**Clinical features**	**Laboratory features**
*Mild*	Abdominal distension/discomfort	No important alterations
	Mild nausea/vomiting	
	Diarrhea	
	Enlarged ovaries	
*Moderate*	Mild features +	Elevated hematocrit (>41 %)
	Ultrasonographic evidence of ascites	Elevated WBC (>15000)
		Hypoproteinemia
*Severe*	Mild and moderate features +	Hemoconcentration (Hct >55 %)
	Clinical evidence of ascites	WBC >25000
	Hydrothorax	CrCl <50 mL/min
	Severe dyspnea	Cr >1.6
	Oliguria/anuria	Na^+^ <135 mEq/L
	Intractable nausea/vomiting	K^+^ >5 mEq/L
	Tense ascites	Elevated liver enzymes
	Low blood/central venous pressure	
	Rapid weight gain (>1 kg in 24 hours)	
	Syncope	
	Severe abdominal pain	
	Venous thrombosis	
*Critical*	Anuria/acute renal failure	Worsening of findings
	Arrhythmia	
	Thromboembolism	
	Pericardial effusion	
	Massive hydrothorax	
	Arterial thrombosis	
	Adult respiratory distress syndrome	
	Sepsis	

Cr = serum creatinine level; CrCl = creatinine clearance; WBC = white blood cell count.

OHSS can be “early” or “late” based on the source of hCG. Early OHSS occurs in the luteal phase of COS after the administration of exogenous hCG to induce oocyte maturation. Late OHSS occurs when ART results in pregnancy and is the consequence of an increase in endogenous hCG levels following conception. In most cases, OHSS is self-limiting and resolves spontaneously within several days. However, OHSS may persist, particularly late OHSS due to pregnancy.

### Risk factors/biomarkers for OHSS

Several primary and secondary risk factors for OHSS have been identified (Table 2). However, their sensitivity and specificity for predicting hyper-response/OHSS is variable [10,11]. Despite this, as indicators of risk, these risk factors/biomarkers assist in the identification of patients that require individualized COS (iCOS).

**Table 2:** **Risk factors/predictive factors for OHSS** (adapted from Humaidan et al [10])

**Risk factor**	**Threshold of risk**
*Primary risk factors (patient related)*	
· High basal AMH	- >3.36 ng/mL independently predicts OHSS [12]
· Young age	- <33 years predicts OHSS [12]
· Previous OHSS	- Moderate and severe cases, particularly those with hospitalization
· PCO like ovaries	- >24 antral follicles in both ovaries combined
*Secondary risk factors (ovarian response-related)*	
*On day of hCG trigger*	
· High number of medium/large follicles	- ≥13 follicles ≥11 mm in diameter [14] - >11 follicles ≥10 mm in diameter [12]
· High or rapidly rising E2 levels and high number of follicles	- E2 5,000 ng/L and/or ≥18 follicles predictive of severe OHSS [14]
· Number of oocytes retrieved	- >11 predicts OHSS [12]
· VEGF levels	- Not applicable
· Elevated inhibin-B levels	- Elevated levels on day 5 of gonadotropin stimulation, at oocyte retrieval and 3 days before
· hCG administration for LPS	- Not applicable
· Pregnancy (increase in endogenous hCG)	- Not applicable

AFC = antral follicle count; AMH = anti-Müllerian hormone; E2 = estradiol; hCG = human chorionic gonadotropin; LPS = luteal phase support; OHSS = ovarian hyperstimulation syndrome; PCOS = polycystic ovary syndrome; VEGF = vascular endothelial growth factor.

There are a number of well-established primary risk factors for the development of OHSS, including young age, polycystic ovary syndrome (PCOS) – characterized by ultrasound and the ratio of luteinizing hormone (LH) to follicle stimulating hormone (FSH) – and a history of an elevated response to gonadotropins, i.e. prior hyper-response/OHSS [9,10,15]. Studies investigating the impact of low body weight/body mass index (BMI) on the development of OHSS report contradictory results [12,15]. Therefore, body weight/BMI does not currently appear to be a useful marker for increased risk of OHSS. Immunological sensitivity, i.e. hypersensitivity or allergies may also be predictive of OHHS. In a prospective cohort study, patients who developed severe OHSS (n = 18/428) had an increased prevalence of allergies (56 % vs. 21 % in the control group) [16]. While a link between OHSS and allergy is plausible, as the pathophysiological changes in the ovaries during OHSS resemble an overactive inflammatory response, the influence of allergies on the development of OHSS requires further study.

Research has identified additional hormonal biomarkers that may also predict a patient’s response to COS and determine their risk of OHSS. In the very early follicular phase of the cycle, a number of antral follicles (2–10 mm in size) are present that are easily detected by transvaginal ultrasound as their appearance is marked by the formation of a fluid-filled cavity adjacent to the oocyte (the antrum) [11,12]. The number of small antral follicles at the beginning of a cycle is related to age and may reflect the ovarian reserve [11,12]. In a study by Kwee et al, an antral follicle count (AFC) >14 had the highest sensitivity (82 %) and specificity (89 %) to positively predict ovarian hyper-response [13].

Basal Anti-Müllerian hormone (AMH) levels prior to COS have also been shown to be predictive for OHSS [17]. Two recent, prospective, randomized controlled trials (RCTs) in large cohorts demonstrated that basal AMH levels ~ ≥3.5 ng/mL were predictive of hyper-response/OHSS with high sensitivity and specificity [12,18]. Moreover, AMH may be a better predictive marker of excessive ovarian response to COS than age, basal FSH, and estradiol (E2) on the day of hCG administration (see below), and has been shown to be at least as good as AFC [12,17]‐[19]. Furthermore, AMH predicts ovarian response independently of age and PCOS [18].

Activating mutations in the FSH receptor (FSHR) gene have been shown to confer a higher response to FSH and therefore FSHR genotype may predispose women to OHSS [20], Although FSHR genotype cannot predict the risk of iatrogenic OHSS at present, it may be used to predict the severity of the condition. Furthermore, mutations in the bone morphogenic protein-15 (BMP-15) gene may predict ovarian hyper-response and OHSS, but further research is required.

Traditionally, high or rapidly rising serum E2 levels on the day of the hCG trigger, denoting oversensitivity to hCG, was used as a predictor of OHSS [11]. However, high E2 levels alone are poor predictors of OHSS [3,10,11]. The number of follicles in combination with serum E2 levels predicts OHSS with high sensitivity and specificity [14]. Despite E2 levels alone being poor predictors of OHSS, they are often closely monitored and used to drive secondary OHSS prevention strategies.

### Preventing OHSS with iCOS

Prevention of OHSS is a multi-stage process. The key to the primary prevention of OHSS during COS is recognizing risk factors and individualizing the ovarian stimulation protocol appropriately using iCOS [17,21]. iCOS should aim to reduce the cycle cancelation rate and the iatrogenic complications of COS, including OHSS, and is key to improving ART outcomes [18,21]. Based on a retrospective study of 1378 patients, basal FSH, BMI, age and number of follicles <11 mm at screening were reported to be the main predictive factors for ovarian response [22]. The implementation of an algorithm (CONSORT) to include these risk factors has been proposed which would inform the choice of starting gonadotropin dose [23].

Such a personalized approach, where even clomiphene citrate with human menopausal gonadotropin or FSH [24] can have a place [25], allows the appropriate treatment to be selected and adapted for each patient and avoids the increased risks that may result from assigning standardized treatment to patient groups (for example, designating doses by weight category). The use of effective biomarkers could be the ultimate tool to drive iCOS. This could potentially comprise a routine diagnostic test performed before COS to predict ovarian response and facilitate iCOS by determining the required stimulatory gonadotropin dose [10], thereby avoiding possible complications, including OHSS.

The use of AMH, as a biomarker to individualize COS protocols, has been evaluated in a retrospective study of women undergoing ART [26]. The study compared 346 women using conventional COS with 423 women treated using COS protocols tailored to the level of AMH. The analysis reported increased embryo transfer rates (79–87 %, P = 0.002), pregnancy rate per cycle (17.9–27.7 %, P = 0.002) and live birth rate (15.9–23.9 %, P = 0.007) in those women on AMH-tailored protocols compared with conventional COS. The study also reported a fall in the incidence of OHSS (96.9–2.3 %, P = 0.002) and failed fertilization (7.8 %–4.5 %, P = 0.066). In the future, pharmacogenetics could also be used to direct iCOS [20].

Before initiating iCOS, patients at high risk of OHSS can be identified from their risk/biomarker profile and the stimulation protocol can be tailored to their needs through iCOS. If iCOS is not correctly applied then patients are more likely to experience OHSS. To minimize the risk of severe complications, secondary preventative measures are normally applied. Various preventative protocols have been proposed to reduce or minimize the risk of developing OHSS during COS, including *in vitro* oocyte maturation, coasting, decreasing the hCG trigger dose, and using a gonadotropin-releasing hormone agonist (GnRHa) trigger. However, despite the widespread use of these preventative techniques, supporting evidence is limited.

There have been few RCTs that fully evaluate the efficacy and safety of these protocols [10], with different centers tending to favor specific techniques based on their own experience.

A recent Cochrane review concluded that there was no evidence to suggest a benefit of coasting to prevent OHSS compared with no coasting or other interventions, but only four of 16 studies included in the review met the RCT inclusion criteria [27]. Despite the lack of data from RCTs to support its use for the reduction of OHSS [27], coasting has been widely adopted. However, coasting is not an option with the newer, long-acting follicular stimulants, such as the recombinant glycoprotein corifollitropin alfa (ELONVA®; MSD).

Due to its long half-life (65 hours), a single injection of corifollitropin alfa is intended to replace daily gonadotropin injections during the first week of COS [28,29]. In two phase 3 trials investigating corifollitropin alfa (100 or 150 μg) as part of a GnRH antagonist COS protocol, rates of moderate-to-severe OHSS were 3.4–4.1 %, compared with 1.6–2.7 % in patients receiving recombinant FSH [30,31]. Recently, the incidence of moderate-to-severe OHSS in women receiving corifollitropin alfa (150 μg) was shown to be 1.8 % in a multicenter, open-label, uncontrolled phase 3 study using a multidose antagonist protocol. First, second and third COS cycles were started by 682, 375 and 198 patients, respectively. OHSS was reported in 24 patients (3.5 %) in the first COS cycle and in seven patients (1.9 %) in the second cycle; it did not occur during the third treatment cycle. A total of 15 cases of mild OHSS were reported; eight cases were considered moderate and another eight were classed as severe OHSS [32]. As OHSS occurred despite the study design excluding patients at high risk of OHSS, this may be indicative of idiosyncrasies in patient management protocols and individual clinical practice.

Metformin has also been used for the prevention of OHSS. In a meta-analysis of eight randomized controlled trials of women with PCOS, metformin given 2 months before starting COS significantly reduced the risk of severe OHSS (odds ratio [OR] of 0.21, 95 % confidence interval [CI] 0.11–0.41, P < 0.00001) [33].

### Cancelling ovulation induction

As OHSS is associated with hCG, terminating the ovulation cycle by cancelling the hCG trigger in the presence of several risk factors for OHSS is the most effective technique to prevent OHSS [15]. hCG induces the production of VEGF, the primary mediator of OHSS [5]. However, this course of action is costly and psychologically demanding for the participants. Therefore, it is usually reserved for patients at high risk of OHSS and those with total loss of cycle control.

*In vitro* oocyte maturation, where immature oocytes are retrieved and matured *in vitro* before fresh embryo transfer, is also an option in these patients. In 56 patients with high risk of OHSS during the controlled ovarian hyperstimulation cycle, hCG was given when the leading follicle reached 12–14 mm in diameter [34]. Seventy-six percent of oocytes matured. All patients underwent fresh embryo transfers, resulting in a clinical pregnancy rate of 46 %. There were no severe cases of OHSS. However, it is worth noting that *in vitro* maturation of oocytes remains an experimental procedure, used only in a small number of clinics around the world.

### Coasting: withholding exogenous gonadotropins

Coasting is the concept of withholding exogenous gonadotropins and postponing the hCG trigger until a patient’s E2 level has declined to a ”safer” pre-defined level (usually ~3000 pg/nL [35,36]).

Follicular size generally correlates with the FSH threshold and, therefore, larger follicles that are more resistant to apoptosis and atresia should continue to grow when FSH levels are declining [35]. Coasting leads to the selective regression of the pool of immature (small/medium) follicles, thereby reducing the functioning granulosa cell mass available for luteinization and resulting in a decline in vasoactive substances involved in the pathogenesis of OHSS, including VEGF (Figure 1[37,38]). Coasting has been shown to reduce the incidence of OHSS in high-risk patients without affecting cycle outcome, as demonstrated by anecdotal data and data from non-randomized trials [3,36,38]‐[40]. However, coasting is becoming less of an option with the newer, long-acting follicular stimulants, such as the recombinant glycoprotein corifollitropin alfa (ELONVA®; MSD) [36,38,39].16 % of patients had ascites and 2.5 % required hospitalization in a systematic review of 12 studies involving 493 patients, only one of which was a RCT [40]. In addition, there are reports that coasting for more than 3–4 days results in lower than anticipated pregnancy and implantation rates [35,36,39].

### Individualizing the hCG trigger dose

Theoretically, decreasing the standard dose of hCG administered to trigger oocyte maturation (10000 IU) might prevent OHSS. Doses of hCG as low as 3300 IU have been shown to effectively trigger oocyte maturation in ART without adversely affecting cycle outcome; 2000 IU was ineffective [41]. Doses of hCG as low as 2500 IU have been shown to be effective in patients with PCOS. However, the benefit of low-dose hCG for the prevention of OHSS is not clear, as data are sparse and the studies that have been conducted comprised small sample sizes, involved a small number of cycles or were not powered to detect a difference in OHSS rate. Importantly, there appears to be no difference between the incidence of severe OHSS with recombinant hCG compared with urinary hCG [42].

### Choice of luteal phase support

A recent Cochrane review has shown that the choice of luteal phase support is related to the incidence of OHSS [43]. This review included a comparison of the use of progesterone versus hCG and progesterone, for luteal phase support, and showed an increased risk of OHSS in the groups taking hCG and progesterone (Peto OR 0.45, 95 % CI 0.26–0.79). The review concluded that the use of hCG should be avoided.

### Employing a dopamine agonist

Recent evidence also demonstrates that the administration of a dopamine agonist, such as cabergoline or guinagolide, from the day of hCG trigger can reduce the incidence of OHSS by inhibiting the phosphorylation of VEGFR-2 in response to hCG [5,44]. To date, two randomized controlled trials comparing the use of cabergoline with intravenous albumin alone have shown that cabergoline (0.5 mg/d) was more effective than albumin in preventing OHSS [45,46]. In addition, one study has shown that women with PCOS are less responsive to cabergoline compared with those without PCOS, most probably due to a decreased production of dopamine and dopamine receptor expression [47]. Interestingly, dopamine agonists cannot prevent late OHSS [5].

### Employing a GnRH agonist trigger

The risk of OHSS can be reduced by using a GnRHa trigger, instead of an hCG trigger, in patients undergoing COS with a GnRH-antagonist protocol. Since the technique was first suggested in 1988 [48], a number of studies have investigated the efficacy and safety of a GnRHa trigger. An analysis by Humaidan and colleagues of three early RCTs demonstrated similar results in patients receiving a GnRHa trigger and those receiving an hCG trigger in terms of number of ocytes retrieved, fertilization rate and embryo quality score [49]. However, patients receiving a GnRHa trigger had poor clinical outcomes, with a reduced likelihood of pregnancy and an extremely high early pregnancy loss rate, which was attributed to luteal phase insufficiency, despite standard luteal phase support (LPS) [49]. Humaidan et al also undertook an analysis of six subsequent RCTs using modified LPS, which yielded similar outcomes in patients receiving GnRHa or hCG triggers, with a non-significant 6 % difference in delivery rate in favor of an hCG trigger [49]. Importantly, the use of a GnRHa trigger completely eliminated OHSS in the 375 women randomized to receive it across all nine RCTs [49], although there are isolated reports of OHSS in patients receiving a GnRHa trigger, particularly in those receiving adjuvant low-dose hCG for LPS, as would be expected [50,51]. LPS strategies in patients receiving a GnRHa trigger are reviewed in detail by Engmann et al [50].

A recent Cochrane review of 11 RCTs concluded that GnRHa should not be routinely used to trigger oocyte maturation due to lower live birth rates and ongoing pregnancy rates, but makes an exception for women at high risk of OHSS, after appropriate counseling [52]. Importantly, this review reported that there were no OHSS events in the GnRHa arm of the study, a result which compares favorably against other preventive strategies. It is possible therefore, that combining GnRHa with embryo vitrification has the potential to provide a good clinical outcome [52].

While both the analysis by Humaidan et al and the Cochrane review both support the use of a GnRHa trigger to prevent OHSS, it should be noted that the Cochrane review included all RCTs employing a GnRHa trigger, irrespective of the LPS used [44]. In contrast, the analysis by Humaidan et al analyzed the RCTs according to LPS, and clearly demonstrated no adverse effect on cycle outcome in patients receiving a GnRH trigger with appropriate LPS [49]. Therefore, GnRHa now appears to be a valid alternative to an hCG trigger for final oocyte maturation.

### Intravenous fluids at time of oocyte retrieval

Albumin has both osmotic and transport functions, properties that underscore its potential for the prevention of OHSS [53]. Conflicting data are available regarding the potential benefit of intravenous (IV) albumin at the time of oocyte retrieval to prevent OHSS. An early Cochrane review of five RCTs clearly showed a benefit associated with the administration of IV albumin at the time of oocyte retrieval in patients at high risk of OHSS, with no effect on pregnancy rate [54]. However, a recent update to this review including eight RCTs concluded that there was limited evidence for the benefit of IV albumin in this setting, although there was no detrimental effect on pregnancy rate [53]. Another recent systematic review and meta-analysis of eight RCTs (seven common to both analyses) made similar conclusions. In contrast, a further systematic review and meta-analysis of nine RCTs found that while there was no statistical benefit regarding the rate of OHSS compared with saline/no fluids, IV albumin significantly reduced pregnancy rates (relative risk 0.85, 95 % CI 0.74–0.98 [55]).

Hydroxyethyl starch (HES) is a plasma expander and a possible alternative to albumin in this setting. As a non-biological substance, HES is not associated with the potential for viral transmission that may be present with albumin [56]. The recent Cochrane review of studies using IV albumin also analyzed the effects of HES at the time of oocyte retrieval in patients at high risk of OHSS in three RCTs [53]. HES was associated with a significant reduction in the incidence of OHSS (OR 0.12, 95 % CI 0.04–0.40), without affecting pregnancy rates.

### Cryopreservation of oocytes and embryos

Cryopreservation is considered a traditional approach for the prevention of OHSS in COS. Oocyte retrieval and elective cryopreservation with subsequent transfer in an unstimulated cycle eliminates further hCG exposure in the active cycle, avoiding the frustration of cycle cancellation and preserving the chance for a live birth. The pregnancy rates achieved with frozen oocytes and embryos are now similar to those achieved in fresh cycles [57].

Cryopreservation appears to reduce, but not eliminate, OHSS without adversely affecting pregnancy rates [58]‐[62]. Cryopreservation has been shown to offer a higher cumulative pregnancy rate than coasting to avoid OHSS [60]. However, variations in policies exist regarding which stage (pronucleate, cleavage stage or blastocyst) and protocol should be used for cryopreservation, and at which stage these should be thawed and transferred [63]. This procedure is however not without risk. In a retrospective review of maternal death related to IVF carried out in the Netherlands, three women with OHSS died following ovum retrieval for cryopreservation. Two women died as a result of adult respiratory distress syndrome and multi-organ failure, and one due cerebrovascular thrombosis. All three patients had all their embryos frozen because they were exhibiting symptoms of OHSS [64].

Recently, interest in cryopreservation has increased due to vitrification, an efficient method of cryopreservation that result in better survival after thawing, due to reduced cellular damage compared with traditional cryopreservation techniques [57]. Vitrification is the rapid process of turning a liquid into an amorphous “glass-like” substance, rather than changing it to a solid by crystallization (i.e. the passage of a liquid to a solid without the intermediate formation of ice crystals). Studies have shown that vitrification is associated with better ART outcomes than slow cooling [65]. Emergency vitrification of embryos has been shown to be successful for the prevention of OHSS in high-risk women [66]. Furthermore, vitrified-warmed blastocyst transfer cycles (n = 136) yielded higher implantation and pregnancy rates than fresh blastocyst transfer cycles (n = 110) in a retrospective study in China [67]. These results prompted the authors to propose a new embryo transfer strategy avoiding fresh transfers altogether and instead vitrifying all blastocysts for warming and transfer in a subsequent cycle. Similarly, in Germany a pregnancy rate of 36.9 % was achieved with vitrification, three times higher than that achieved with traditional cryopreservation in the same centre, which led the investigators to ask: “is it still fair to advocate a slow freezing rate?” [68]. Given the widespread use of traditional cryopreservation for the prevention of OHSS, and the limited but promising results with vitrification published so far, it seems likely that vitrification will take over where traditional cryopreservation has led.

### Treatment of OHSS

If iCOS is not applied and risk reduction strategies are unsuccessful, measures are required to minimize the effect of OHSS and prevent further morbidity. Mild OHSS, which due to the very nature of COS occurs in most patients, and moderate OHSS with no clinical evidence of ascites or enlarged ovaries are not associated with complications and as a result do not require specific treatment. Mild OHSS and moderate OHSS can be treated symptomatically and patients monitored on an outpatient basis [69], for example, by tracking weight gain, which is one of the first signs of fluid retention. Severe OHSS, on the other hand, must be regarded as a potentially fatal complication that requires immediate treatment to maintain circulatory volume and restore electrolyte balance using IV fluids [69]. However, this often leads to increased ascitic fluid formation [70]. Historically, the treatment of OHSS, comprising IV therapy with or without paracentesis (aspiration of the ascitic fluid), involved prolonged hospitalization [69]‐[71]. Aggressive outpatient management of patients with moderate-to-severe OHSS using early paracentesis has been shown to effectively reduce the need for hospitalization [70]‐[73]. Both abdominal [70,71] and transvaginal [72,73] routes for paracentesis have been shown to be effective. Furthermore, early outpatient paracentesis for moderate-to-severe OHSS is more cost effective than traditional conservative inpatient therapy [74].

In patients with moderate OHSS, aggressive early paracentesis can prevent the progression of disease severity [75]. In addition to preventing hospitalization, paracentesis rapidly relieves symptoms, with patients experiencing improvements in urine output, renal function and hematocrit levels as early as 24 hours following the procedure [75]. Ultrasound-guided transvaginal aspiration of ascitic fluid has also been shown to be safe and effective in improving symptoms, preventing complications, and shortening the hospital stay for women with severe OHSS [76,77]. It has also been shown that early (luteal phase) transvaginal aspiration of accumulating fluid in patients developing intra-abdominal ascites can reduce the need for hospitalization [71,78].

A recent review of the clinical aspects of OHSS provides detailed recommendations for management according to patient diagnosis and risk [79].

## Conclusions

Prevention of OHSS begins with tailoring an individual’s ovarian stimulation protocol based on their risk profile, through iCOS. Selecting one standardized preventative approach for all patients or a large cohort of patients undergoing COS is challenging, because the benefits and risks associated with each strategy vary between individuals. Identification of hormonal, functional and genetic markers of ovarian response will facilitate iCOS. Indeed, if the risk factors and biomarkers for OHSS are recognized and patients are correctly treated with iCOS, OHSS may no longer be an issue. In the meantime, despite the scarcity of data from RCTs to support their use, there are various secondary preventative measures that can be employed to reduce the risk of OHSS once COS has begun, including canceling the cycle, coasting, individualizing the hCG trigger dose or using a GnRH trigger (for those using a GnRH antagonist protocol), the use of IV fluids at the time of oocyte retrieval, and cryopreserving/vitrifying all oocytes.

## Abbreviations

AFC: Antral follicle count; AMH: Anti-Müllerian hormone; ART: Assisted reproductive technology; BMI: Body mass index; BMP-15: Bone morphogenic protein-15; CONSORT: Consistency in r-FSH starting doses for individualized treatment; COS: Controlled ovarian stimulation; Cr: Serum creatinine level; CrCl: Creatinine clearance; E2: Estradiol; FSH: Follicle stimulating hormone; FSHR: Follicle stimulating hormone receptor; GnRH: Gonadotropin-releasing hormone; GnRHa: Gonadotropin-releasing hormone agonist; hCG: Human chorionic gonadotropin; Hct: Hemoconcentration; HES: Hydroxyethyl starch; iCOS: Individualized controlled ovarian stimulation; IV: Intravenous; LH: Luteinizing hormone; LPS: Luteal phase support; OHSS: Ovarian hyperstimulation syndrome; PCOS: Polycystic ovary syndrome; RCTs: Randomized controlled trials; VEGF: Vascular endothelial growth factor; VEGFR-2: Vascular endothelial growth factor receptor 2; WBC: White blood cell count.

## Competing interests

KF has no competing interests to declare. DE is an employee of Merck Serono S.A. – Geneva, Switzerland (Global Scientific Director, Fertility and Endocrinology Business Unit).

## Authors’ contributions

KF and DE contributed to the writing and editing of this manuscript and approved the final version for submission. They are fully and equally responsible for the content of the article. Both authors read and approved the final manuscript.

## References

[B1] de Mouzon J Goossens V Bhattacharya S Castilla JA Ferraretti AP Korsak V Kupka M Nygren KG Nyboe Andersen A European IVF-monitoring (EIM) Consortium, for the European Society of Human Reproduction and Embryology (ESHRE) Assisted reproductive technology in Europe, 2006: results generated from European registers by ESHRE Hum Reprod 2010 25 1851 1862 10.1093/humrep/deq124 20570973

[B2] Klemetti R Sevón T Gissler M Hemminki E Complications of IVF and ovulation induction Hum Reprod 2005 20 3293 3300 10.1093/humrep/dei253 16126753

[B3] Alper MM Smith LP Sills ES Ovarian hyperstimulation syndrome: current views on pathophysiology, risk factors, prevention, and management J Exp Clin Assist Reprod 2009 10 3 20485578 PMC2868304

[B4] Brinsden PR Wada I Tan SL Balen A Jacobs HS Diagnosis, prevention and management of ovarian hyperstimulation syndrome Br J Obstet Gynaecol 1995 102 767 772 10.1111/j.1471-0528.1995.tb10840.x 7547731

[B5] Gómez R Soares SR Busso C Garcia-Velasco JA Simón C Pellicer A Physiology and pathology of ovarian hyperstimulation syndrome Semin Reprod Med 2010 28 448 457 10.1055/s-0030-1265670 21082502

[B6] Nastri CO Ferriani RA Rocha IA Martins WP Ovarian hyperstimulation syndrome: pathophysiology and prevention J Assist Reprod Genet 2010 27 121 128 10.1007/s10815-010-9387-6 20140640 PMC2842872

[B7] Soares SR Gómez R Simón C García-Velasco JA Pellicer A Targeting the vascular endothelial growth factor system to prevent ovarian hyperstimulation syndrome Hum Reprod Update 2008 14 321 333 10.1093/humupd/dmn008 18385260

[B8] Delvigne A Rozenberg S Review of clinical course and treatment of ovarian hyperstimulation syndrome (OHSS) Hum Reprod Update 2003 9 77 96 10.1093/humupd/dmg005 12638783

[B9] Navot D Bergh PA Laufer N Ovarian hyperstimulation syndrome in novel reproductive technologies: prevention and treatment Fertil Steril 1992 58 249 261 1633889 10.1016/s0015-0282(16)55188-7

[B10] Humaidan P Quartarolo J Papanikolaou EG Preventing ovarian hyperstimulation syndrome: guidance for the clinician Fertil Steril 2010 94 389 400 10.1016/j.fertnstert.2010.03.028 20416867

[B11] Papanikolaou EG Humaidan P Polyzos NP Tarlatzis B Identification of the high-risk patient for ovarian hyperstimulation syndrome Semin Reprod Med 2010 28 458 462 10.1055/s-0030-1265671 21082503

[B12] Lee TH Liu CH Huang CC Wu YL Shih YT Ho HN Yang YS Lee MS Serum anti-Müllerian hormone and estradiol levels as predictors of ovarian hyperstimulation syndrome in assisted reproduction technology cycles Hum Reprod 2008 23 160 167 18000172 10.1093/humrep/dem254

[B13] Kwee J Elting ME Schats R McDonnell J Lambalk CB Ovarian volume and antral follicle count for the prediction of low and hyper responders with in vitro fertilization Reprod Biol Endocrinol 2007 5 9 10.1186/1477-7827-5-9 17362511 PMC1847823

[B14] Papanikolaou EG Pozzobon C Kolibianakis EM Camus M Tournaye H Fatemi HM Van Steirteghem A Devroey P Incidence and prediction of ovarian hyperstimulation syndrome in women undergoing gonadotropin-releasing hormone antagonist in vitro fertilization cycles Fertil Steril 2006 85 112 120 10.1016/j.fertnstert.2005.07.1292 16412740

[B15] Delvigne A Rozenberg S Epidemiology and prevention of ovarian hyperstimulation syndrome (OHSS): a review Hum Reprod 2002 8 559 577 10.1093/humupd/8.6.559 12498425

[B16] Enskog A Henriksson M Unander M Nilsson L Brannstrom M Prospective study of the clinical and laboratory parameters of patients in whom ovarian hyperstimulation syndrome developed during controlled ovarian hyperstimulation for in vitro fertilization Fertil Steril 1999 71 808 814 10.1016/S0015-0282(99)00090-4 10231037

[B17] La Marca A Sighinolfi G Radi D Argento C Baraldi E Artenisio AC Stabile G Volpe A Anti-Mullerian hormone (AMH) as a predictive marker in assisted reproductive technology (ART) Hum Reprod Update 2010 16 113 130 10.1093/humupd/dmp036 19793843

[B18] Nardo LG Gelbaya TA Wilkinson H Roberts SA Yates A Pemberton P Laing I Circulating basal anti-Müllerian hormone levels as predictor of ovarian response in women undergoing ovarian stimulation for in vitro fertilization Fertil Steril 2009 92 1586 1593 10.1016/j.fertnstert.2008.08.127 18930213

[B19] Broer SL Dólleman M Opmeer BC Fauser BC Mol BW Broekmans FJ AMH and AFC as predictors of excessive response in controlled ovarian hyperstimulation: a meta-analysis Hum Reprod Update 2011 17 46 54 10.1093/humupd/dmq034 20667894

[B20] Rizk B Symposium: Update on prediction and management of OHSS, Genetics of ovarian hyperstimulation syndrome Reprod Biomed Online 2009 19 14 27 10.1016/S1472-6483(10)60041-7 19573286

[B21] Bosch E Ezcurra D Individualised controlled ovarian stimulation (iCOS): maximising success rates for assisted reproductive technology patients Reprod Biol Endocrinol 2011 9 82 10.1186/1477-7827-9-82 21693025 PMC3150250

[B22] Howles CM Saunders H Alam V Engrand P Predictive factors and a corresponding treatment algorithm for controlled ovarian stimulation in patients treated with recombinant human follicle stimulating hormone (follitropin alfa) during assisted reproduction technology (ART) procedures. An analysis of 1378 patients Curr Med Res Opin 2006 22 907 918 10.1185/030079906X104678 16709312

[B23] Olivennes F Howles CM Borini A Germond M Trew G Wikland M Zegers-Hochschild F Saunders H Alam V Individualizing FSH dose for assisted reproduction using a novel algorithm: the CONSORT study Reprod Biomed Online 2009 18 195 204 10.1016/S1472-6483(10)60256-8 21575853

[B24] Bayly CM McBain JC Clarke GA Gronow MJ Johnston WI Martin MJ Speirs AL Ovarian stimulation regimens in an in vitro fertilization program: a comparative analysis Ann N Y Acad Sci 1985 442 123 127 10.1111/j.1749-6632.1985.tb37512.x 3925832

[B25] Fiedler K Ludwig M Use of clomiphene citrate in in vitro fertilization (IVF) and IVF/intracytoplasmic sperm injection cycles Fertil Steril 2003 80 1521 1523 10.1016/S0015-0282(03)02208-8 14667897

[B26] Yates AP Rustamov O Roberts SA Lim HY Pemberton PW Smith A Nardo LG Anti-Mullerian hormone-tailored stimulation protocols improve outcomes whilst reducing adverse effects and costs of IVF Obstet Gynecol Survey 2011 66 760 776 10.1097/OGX.0b013e318240254e 21672928

[B27] D'Angelo A Brown J Amso NN 2011 Cochrane Database Syst Rev 2011 6 CD002811 21678336 10.1002/14651858.CD002811.pub3

[B28] Devroey P Fauser BC Platteau P Beckers NG Dhont M Mannaerts BM Induction of multiple follicular development by a single dose of long-acting recombinant follicle-Stimulating hormone (FSH-CTP, corifollitropin alfa) for controlled ovarian stimulation before in vitro fertilization J Clin Endocrinol Metab 2004 89 2062 2070 10.1210/jc.2003-031766 15126522

[B29] Fauser BC Alper MM Ledger W Schoolcraft WB Zandvliet A Mannaerts BM Engage Investigators: Pharmacokinetics and follicular dynamics of corifollitropin alfa versus recombinant FSH during ovarian stimulation for IVF Reprod Biomed Online 2010 21 593 601 10.1016/j.rbmo.2010.06.032 20843746

[B30] The corifollitropin alfa Ensure Study Group Corifollitropin alfa for ovarian stimulation in IVF: a randomized trial in lower-body-weight women Reprod Biomed Online 2010 21 66 76 20483664 10.1016/j.rbmo.2010.03.019

[B31] Devroey P Boostanfar R Koper NP Mannaerts BM Ijzerman-Boon PC Fauser BC ENGAGE Investigators A double-blind, non-inferiority RCT comparing corifollitropin alfa and recombinant FSH during the first seven days of ovarian stimulation using a GnRH antagonist protocol Hum Reprod 2009 24 3063 3072 10.1093/humrep/dep291 19684043 PMC2777786

[B32] Norman RJ Zegers-Hochschild F Salle BS Elbers J Heijnen E Marintcheva-Petrova M Mannaerts B Trust Investigators: Repeated ovarian stimulation with corifollitropin alfa in patients in a GnRH antagonist protocol: no concern for immunogenicity Hum Reprod 2011 26 2200 2208 10.1093/humrep/der163 21622693 PMC3137390

[B33] Costello MF Chapman M Conway U A systematic review and meta-analysis of randomized controlled trials on metformin co-administration during gonadotrophin ovulation induction or IVF in women with polycystic ovary syndrome Hum Reprod 2006 21 1387 1399 10.1093/humrep/dei501 16449310

[B34] Lim K Lee W Lim J IVM after interruption of COH for the prevention of OHSS Fertil Steril 2005 84 S84 S85

[B35] Abdallah R Kligman I Davis O Rosenwaks Z Withholding gonadotropins until human chorionic gonadotropin administration Semin Reprod Med 2010 28 486 492 10.1055/s-0030-1265675 21082507

[B36] Levinsohn-Tavor O Friedler S Schachter M Raziel A Strassburger D Ron-El R Coasting-what is the best formula? Hum Reprod 2003 18 937 940 10.1093/humrep/deg230 12721165

[B37] Tozer AJ Iles RK Iammarrone E Gillott CM Al-Shawaf T Grudzinskas JG The effects of 'coasting' on follicular fluid concentrations of vascular endothelial growth factor in women at risk of developing ovarian hyperstimulation syndrome Hum Reprod 2004 19 522 528 10.1093/humrep/deh096 14998945

[B38] García-Velasco JA Zúñiga A Pacheco A Gómez R Simón C Remohí J Pellicer A Coasting acts through downregulation of VEGF gene expression and protein secretion Hum Reprod 2004 19 1530 1538 10.1093/humrep/deh298 15155606

[B39] Mansour R Aboulghar M Serour G Amin Y Abou-Setta AM Criteria of a successful coasting protocol for the prevention of severe ovarian hyperstimulation syndrome Hum Reprod 2005 20 3167 3172 10.1093/humrep/dei180 16006465

[B40] Delvigne A Rozenberg S A qualitative systematic review of coasting, a procedure to avoid ovarian hyperstimulation syndrome in IVF patients Hum Reprod Update 2002 8 269 291 10.1093/humupd/8.3.291 12078839

[B41] Kashyap S Parker K Cedars MI Rosenwaks Z Ovarian hyperstimulation syndrome prevention strategies: reducing the human chorionic gonadotropin trigger dose Semin Reprod Med 2010 28 475 485 10.1055/s-0030-1265674 21082506

[B42] Youssef MA Al-Inany HG Aboulghar M Mansour R Abou-Setta AM Recombinant versus urinary human chorionic gonadotrophin for final oocyte maturation triggering in IVF and ICSI cycles Cochrane Database Syst Rev2011b 4 CD003719 21491386 10.1002/14651858.CD003719.pub3

[B43] van der Linden M Buckingham K Farquhar C Kremer JA Metwally M Luteal phase support for assisted reproduction cycles.Cochrane.Database Syst. Rev 2011 10 CD009154 10.1002/14651858.CD009154.pub2 21975790

[B44] Youssef MA van Wely M Hassan MA Al-Inany HG Mochtar M Khattab S van der Veen F Can dopamine agonists reduce the incidence and severity of OHSS in IVF/ICSI treatment cycles? A systematic review and meta-analysis Hum Reprod Update 2010 16 459 466 10.1093/humupd/dmq006 20354100

[B45] Tehraninejad ES Hafezi M Arabipoor A Aziminekoo E Chehrazi M Bahmanabadi A Comparison of cabergoline and intravenous albumin in the prevention of ovarian hyperstimulation syndrome: a randomized clinical trial J Assist Reprod Genet 2010 29 259 264 22231013 10.1007/s10815-011-9708-4 PMC3288141

[B46] Carizza C Abdelmassih V Abdelmassih S Ravizzini P Salgueiro L Salgueiro PT Jine LT Nagy P Abdelmassih R Cabergoline reduces the early onset of ovarian hyperstimulation syndrome: a prospective randomized study Reprod Biomed Online 2008 17 751 755 10.1016/S1472-6483(10)60401-4 19079957

[B47] Gomez R Ferrero H Gado-Rosas F Gaytan M Morales C Zimmermann RC Simon C Gaytan F Pellicer A Evidences for the existence of a low dopaminergic tone in polycystic ovarian syndrome: implications for OHSS development and treatment J Clin Endocrinol Metab 2011 96 2484 2492 10.1210/jc.2011-0075 21646367

[B48] Itskovitz J Boldes R Barlev A Erlik Y Kahana L Brandes JM The induction of LH surge and oocyte maturization by GnRH analogue (buserelin) in women undergoing ovarian stimulation for in vitro fertilization Gynecol Endocrinol 1988 2 Suppl 2 1653055821

[B49] Humaidan P Kol S Papanikolaou E on behalf of the ‘The Copenhagen GnRH Agonist Triggering Workshop Group’ GnRH agonist for triggering of final oocyte maturation: time for a change of practice? Hum Reprod Update 2011 17 510 524 10.1093/humupd/dmr008 21450755

[B50] Engmann L Benadiva C Ovarian hyperstimulation syndrome prevention strategies: Luteal support strategies to optimize pregnancy success in cycles with gonadotropin-releasing hormone agonist ovulatory trigger Semin Reprod Med 2010 28 506 512 10.1055/s-0030-1265678 21082510

[B51] Kol S Itskovitz-Eldor J Gonadotropin-releasing hormone agonist trigger: the way to eliminate ovarian hyperstimulation syndrome -a 20-year experience Semin Reprod Med 2010 28 500 505 10.1055/s-0030-1265677 21082509

[B52] Youssef MA Van der Veen F Al-Inany HG Griesinger G Mochtar MH van Wely M Gonadotropin-releasing hormone agonist versus HCG for oocyte triggering in antagonist assisted reproductive technology cycles Cochrane Database Syst Rev 2011 1 CD008046 21069701 10.1002/14651858.CD008046.pub2

[B53] Youssef MA Al-Inany HG Evers JL Aboulghar M Intra-venous fluids for the prevention of severe ovarian hyperstimulation syndrome Cochrane Database Syst Rev2011a 2 CD001302 21328249 10.1002/14651858.CD001302.pub2

[B54] Aboulghar M Evers JH Al-Inany H Intravenous albumin for preventing severe ovarian hyperstimulation syndrome: a Cochrane review Hum Reprod 2002 17 3027 3032 10.1093/humrep/17.12.3027 12456597

[B55] Jee BC Suh CS Kim YB Kim SH Choi YM Kim JG Moon SY Administration of intravenous albumin around the time of oocyte retrieval reduces pregnancy rate without preventing ovarian hyperstimulation syndrome: a systematic review and meta-analysis Gynecol Obstet Invest 2010 70 47 54 10.1159/000286379 20173327

[B56] Gokmen O Ugur M Ekin M Keles G Turan C Oral H Intravenous albumin versus hydroxyethyl starch for the prevention of ovarian hyperstimulation in an in-vitro fertilization programme: a prospective randomized placebo controlled study Eur J Obstet Gynecol Reprod Biol 2001 96 187 192 10.1016/S0301-2115(00)00452-8 11384805

[B57] Herrero L Martínez M Garcia-Velasco JA Current status of human oocyte and embryo cryopreservation Curr Opin Obstet Gynecol 2011 23 245 250 21734500 10.1097/GCO.0b013e32834874e2

[B58] Sills ES McLoughlin LJ Genton MG Walsh DJ Coull GD Walsh AP Ovarian hyperstimulation syndrome and prophylactic human embryo cryopreservation: analysis of reproductive outcome following thawed embryo transfer J Ovarian Res 2008 1 7 10.1186/1757-2215-1-7 19014420 PMC2585559

[B59] Fitzmaurice GJ Boylan C McClure N Are pregnancy rates compromised following embryo freezing to prevent OHSS? Ulster Med J 2008 77 164 167 18956797 PMC2604472

[B60] Gera PS Tatpati LL Allemand MC Wentworth MA Coddington CC Ovarian hyperstimulation syndrome: steps to maximize success and minimize effect for assisted reproductive outcome Fertil Steril 2010 94 173 178 10.1016/j.fertnstert.2009.02.049 19356753

[B61] Queenan JT Veeck LL Toner JP Oehninger S Muasher SJ Cryopreservation of all prezygotes in patients at risk of severe hyperstimulation does not eliminate the syndrome, but the chances of pregnancy are excellent with subsequent frozen-thaw transfers Hum Reprod 1997 12 1573 1576 10.1093/humrep/12.7.1573 9262299

[B62] Ferraretti AP Gianaroli L Magli C Fortini D Selman HA Feliciani E Elective cryopreservation of all pronucleate embryos in women at risk of ovarian hyperstimulation syndrome: efficiency and safety Hum Reprod 1999 14 1457 1460 10.1093/humrep/14.6.1457 10357958

[B63] D'Angelo A Ovarian hyperstimulation syndrome prevention strategies: cryopreservation of all embryos Semin Reprod Med 2010 28 513 518 10.1055/s-0030-1265679 21082511

[B64] Braat DD Schutte JM Bernardus RE Mooij TM van Leeuwen FE Maternal death related to IVF in the Netherlands 1984–2008 Hum Reprod 2010 25 1782 1786 10.1093/humrep/deq080 20488805

[B65] Busso CE Garcia-Velasco JA Simon C Pellicer A Prevention of OHSS: Current strategies and new insights Middle East Fertil Soc J 2010 15 223 230 10.1016/j.mefs.2010.06.013

[B66] Selman H Brusco GF Fiorini F Barnocchi N Mariani M El-Danasouri I Vitrification is a highly efficient method to cryopreserve human embryos in in vitro fertilization patients at high risk of developing ovarian hyperstimulation syndrome Fertil Steril 2009 91 4 Suppl 1611 1613 19200960 10.1016/j.fertnstert.2008.12.027

[B67] Zhu D Zhang J Cao S Zhang J Heng BC Huang M Ling X Duan T Tong GQ Vitrified-warmed blastocyst transfer cycles yield higher pregnancy and implantation rates compared with fresh blastocyst transfer cycles-time for a new embryo transfer strategy? Fertil Steril 2011 95 1691 1695 10.1016/j.fertnstert.2011.01.022 21315339

[B68] Al-Hasani S Ozmen B Koutlaki N Schoepper B Diedrich K Schultze-Mosgau A Three years of routine vitrification of human zygotes: is it still fair to advocate slow-rate freezing? Reprod Biomed Online 2007 14 288 293 10.1016/S1472-6483(10)60869-3 17359578

[B69] Aboulghar M Treatment of ovarian hyperstimulation syndrome Semin Reprod Med 2010 28 532 10.1055/s-0030-1265681 21082512

[B70] Shrivastav P Nadkarni P Craft I Day care management of severe ovarian hyperstimulation syndrome avoids hospitalization and morbidity Hum Reprod 1994 9 812 814 7929727 10.1093/oxfordjournals.humrep.a138601

[B71] Smith LP Hacker MR Alper MM Patients with severe ovarian hyperstimulation syndrome can be managed safely with aggressive outpatient transvaginal paracentesis Fertil Steril 2009 92 1953 1959 10.1016/j.fertnstert.2008.09.011 18976762

[B72] Fluker MR Copeland JE Yuzpe AA An ounce of prevention: outpatient management of the ovarian hyperstimulation syndrome Fertil Steril 2000 73 821 824 10.1016/S0015-0282(99)00606-8 10731547

[B73] Lincoln SR Opsahl MS Blauer KL Black SH Schulman JD Aggressive outpatient treatment of ovarian hyperstimulation syndrome with ascites using transvaginal culdocentesis and intravenous albumin minimizes hospitalization J Assist Reprod Genet 2002 19 159 163 10.1023/A:1014828027282 12036082 PMC3455652

[B74] Csokmay JM Yauger BJ Henne MB Armstrong AY Queenan JT Segars JH Cost analysis model of outpatient management of ovarian hyperstimulation syndrome with paracentesis: "tap early and often" versus hospitalization Fertil Steril 2010 93 167 173 10.1016/j.fertnstert.2008.09.054 18990389 PMC3575958

[B75] Grossman LC Michalakis KG Browne H Payson MD Segars JH The pathophysiology of ovarian hyperstimulation syndrome: an unrecognized compartment syndrome Fertil Steril 2010 94 1392 1398 10.1016/j.fertnstert.2009.07.1662 19836016 PMC3124341

[B76] Aboulghar MA Mansour RT Serour GI Sattar MA Amin YM Elattar I Management of severe ovarian hyperstimulation syndrome by ascitic fluid aspiration and intensive intravenous fluid therapy Obstet Gynecol 1993 81 108 111 8416442

[B77] Aboulghar M, Rizk B Ovarian stimulation 2011 Cambridge University Press, Cambridge 123

[B78] Fluker MR Copeland JE Yuzpe AA An ounce of prevention: outpatient management of the ovarian hyperstimulation syndrome Fertil Steril 2000 73 821 824 10.1016/S0015-0282(99)00606-8 10731547

[B79] Shmorgun D Claman P The diagnosis and management of ovarian hyperstimulation syndrome J Obstet Gynaecol Can 2011 11 1156 1162 22082791 10.1016/S1701-2163(16)35085-X

